# Diagnostic role of the fibrosis-4 index and nonalcoholic fatty liver disease fibrosis score as a noninvasive tool for liver fibrosis scoring

**DOI:** 10.1097/MD.0000000000040214

**Published:** 2024-10-25

**Authors:** Mingxi Chen, Chang Guo, Ke Ouyang, Na Liu

**Affiliations:** a Department of Infectious Disease and Liver Disease, The Second Hospital of Nanjing, Nanjing, Jiangsu, China; b Department of Internal Medicine, Shandong Rehabilitation Hospital, Jinan, Shandong, China.

**Keywords:** fibrosis-4 index, liver biopsy, liver fibrosis, nonalcoholic fatty liver disease fibrosis score

## Abstract

Nonalcoholic fatty liver disease (NAFLD) is characterized by liver fibrosis, which serves as a crucial indicator of its progression and prognosis. Owing to the limitations of biopsy, which is the gold standard for measuring liver fibrosis, a reliable and noninvasive marker is required. We evaluated the diagnostic role of the fibrosis-4 (FIB-4) index and nonalcoholic fatty liver disease fibrosis score (NFS) in patients with NAFLD with varying severities of liver fibrosis. The FIB-4 index and NFS were calculated using laboratory data from 121 patients who underwent liver biopsies between January 2022 and December 2023. The results were compared with those of the Scheuer scoring system for liver biopsies (F0, F1 + F2, and F3 + F4) to determine the sensitivity and specificity of the FIB-4 index and the liver disease fibrosis score in detecting and staging liver fibrosis. Twenty-one patients had advanced fibrosis (F3–F4), and 100 had minimal or mild fibrosis (F0–F2). The degree of liver fibrosis increased with decreased albumin, alanine aminotransferase and platelet count levels, and increasing age. Receiver operating characteristic curve analysis for the FIB-4 index and NFS revealed that the areas under the curve for the FIB-4 index and NFS were 0.895 (95% confidence interval: 0.836–0.954) and 0.882 (95% confidence interval: 0.813–0.952), respectively. The FIB-4 indices showed 95.24% sensitivity at a cutoff point of 1.30, and 85% specificity at a cutoff point of 2.67, while the NFS indices showed 95.24% sensitivity at −1.455 cutoff point and 95% specificity at a cutoff point of 0.676. The FIB-4 index and NFS may replace biopsy for the detection of fibrosis in patients with NAFLD.

## 1. Introduction

Nonalcoholic fatty liver disease (NAFLD) is the most common chronic liver disease endangering public health in developed countries.^[1]^ Its spectrum includes nonalcoholic simple fatty liver disease, nonalcoholic steatohepatitis (NASH), and end-stage liver disease caused by liver fibrosis, cirrhosis, and cancer.^[2–4]^ Liver fibrosis and cirrhosis caused by NASH are decisive factors of poor prognosis in patients with NAFLD. Therefore, their importance goes beyond their effects on liver function.^[5]^

Liver biopsy is considered the definitive diagnostic procedure for distinguishing between simple steatosis and NASH within the spectrum of NAFLD. It is also essential for evaluating the extent of fibrosis and determining the stage of the disease.^[6–8]^ Although liver biopsy is recognized as the gold standard for diagnosing liver conditions, its invasive nature introduces certain limitations. The procedure is prone to sampling errors and interobserver variability, which can affect diagnostic accuracy.^[9,10]^ Additionally, the potential for clinical complications, including bleeding, makes it challenging to use liver biopsy for routine long-term monitoring of patients.^[11]^ This has led to the development of alternative noninvasive methods that have been the subject of intensive research over the past decade.^[12]^ For instance, various noninvasive tests have been developed, relying on a combination of clinical and laboratory indicators. These include scoring systems such as the NAFLD fibrosis score (NFS) and the fibrosis-4 (FIB-4) score, as well as imaging techniques like ultrasound-based elastography.^[9,13,14]^ The utility of the FIB-4 index as a screening tool has been studied primarily in patients with hepatitis C and human immunodeficiency virus infections in secondary centers as well as in other highly prevalent populations.^[15]^ NFS is based on laboratory tests and is a prognostic indicator of the risk of developing advanced fibrosis and mortality.^[16]^ Despite their clinical utility, these scoring systems can be influenced by a variety of factors, both hepatic and extrahepatic, such as age, comorbidities, and the prevalence of fibrosis or NASH. Consequently, the accuracy of these scores may be compromised in smaller patient subgroups, potentially leading to misestimations.^[1,17]^ In these scenarios, the reliability of a straightforward clinical scoring system as a diagnostic tool is subject to debate. Therefore, further evaluation is required before definitive conclusions can be drawn.

We aimed to determine the diagnostic roles of FIB-4 and NFS by comparing them with a biopsy assessment of liver fibrosis. This may promote early detection of liver fibrosis in patients.

## 2. Materials and methods

### 2.1. Study design and population

The main purpose of this cross-sectional, noninterventional, retrospective study was to identify liver fibrosis using the FIB-4 index and NFS in patients diagnosed with NAFLD by biopsy-proven between January 2022 and December 2023. We included 121 patients with biopsy-proven NAFLD at The Second Hospital of Nanjing in Jiangsu Province.

### 2.2. Inclusion and exclusion criteria

Patients > 18 years old, with biopsy-proven NAFLD. Patients with heavy alcohol intake (>30 g/d for men and >20 g/d for women), and those with viral hepatitis and other chronic liver diseases (e.g., primary biliary cholangitis, drug-induced liver injury, and autoimmune liver disease), as evidenced by biopsy, were excluded.

### 2.3. Liver histological examination

Liver tissues of at least 10 mm with at least 6 portal veins were eligible. The Scheuer scoring system was used as the histological scoring criterion for liver fibrosis. Liver fibrosis was classified into 5 stages: F0: no fibrosis; F1: enlarged fibrotic portal tracts; F2: periportal or portal–portal septa with an intact architecture; F3: fibrosis with architectural distortion but no obvious cirrhosis; and F4: cirrhosis, probable, or definite.^[16]^

The patients were divided into 3 groups. The first group included patients at the F0 stage according to the Scheuer scoring system (minimal fibrosis). The second group included patients at stages F1 and F2 (mild fibrosis), and the third group comprised patients at stages F3 and F4 (advanced fibrosis).

### 2.4. Fibrosis-4 and nonalcoholic fatty liver disease fibrosis score

The FIB-4 score was calculated using a laboratory data meter, according to the following formula:


$$\begin{array}{l} \begin{array}{lll} & FIB\text{-}4\; & =\left(age\;\left[years\right]\times AST\;\left[U/L\right]\right)/\left(PLT\;\left[10^{9}/L\right]\times \left(ALT\;\left[U/L\right]1/2\right)\right).\; \end{array} \end{array}$$

The NFS was calculated using the following formula:


$$\begin{array}{l} \begin{array}{l} NFS=-1.675+0.037\times age\;\left(year\right)+0.094\times BMI\;\;\;\left(kg/m^{2}\right)\;\\ +1.13\times IFG\;\;\;or\;\;\;TTDM\;\left(Yes=1,\;No=0\right)\;\\ +0.99\times AST\;\left(IU/L\right)/ALT\;\left(IU/L\right)-0.013\times PLT\;\left(10^{9}/L\right)\;\\ -0.66\times ALB\;\left(g/dL\right).\; \end{array} \end{array}$$

BMI is body mass index, ALB is albumin, AST is aspartate transferase, ALT is alanine aminotransferase, IFG is impaired fasting glycemia, PLT is platelet count, and T2DM is type 2 diabetes mellitus.

In the analysis, the FIB-4 index was grouped according to the following cutoff points: <1.30, 1.30 and 2.67, and >2.67. The cutoff points for NFS were <‐1.455, between ‐1.455 and 0.676, and >0.676.

### 2.5. Statistical analysis

The approximate normal distribution of quantitative data was expressed as the mean ± standard deviation and the analysis of variance was used for comparison between groups. Quantitative continuous data with skewed distribution were described using the median and interquartile ranges, and the Kruskal–Wallis test was used for comparison between groups. The chi-square test was used to analyze categorical data. The area under the receiver operating characteristic (ROC) curve, which ranges from 0.50 (no discrimination) to 1.0 (perfect discrimination), was also calculated. The results were analyzed using the IBM SPSS Statistics Version 26 predictive analytics software. A *P*-value < .05 was considered significant. The sensitivity, specificity, positive predictive value, and negative predictive value (NPV) corresponding to the optimal cutoff values were calculated.

## 3. Results

Patients (n = 121; mean age, 46 years; range, 19–81 years) with NAFLD were tested for liver fibrosis using liver biopsy during the study. More females were present than males (58.68% vs 41.32%). As indicated in Section 2, the study population was divided into 3 groups based on the stage of liver fibrosis using the Scheuer system: minimal fibrosis (F0), mild fibrosis (F1 + F2), and advanced fibrosis (F3 + F4). The patient distribution according to the stages of liver fibrosis was as follows: 11 (9.09%) patients with stage F0, 89 (73.55%) with stages F1 and F2, and 21 (17.36%) with stages F3 and F4. The FIB-4 and NFS results were divided into 3 groups (mean FIB-4 index, 2.17; mean NFS index, 0.63) (Table 1). As the degree of liver fibrosis increased, albumin, alanine aminotransferase, and platelet count levels gradually decreased; the differences between the groups were statistically significant (*P* < .05), whereas the degree of liver fibrosis increased with increasing age. Sex, body mass index, aspartate aminotransferase, glucose, and uric acid levels were not significantly different among the 3 groups (Table 2).

**Table 1:** General characteristics of the population.

Characteristic variables	Count (n)	Percentage (%)
Sex		
Male	50	41.32
Female	71	58.68
Diabetes mellitus		
Yes	17	14.05
No	104	85.95
Hypertension		
Yes	29	23.97
No	92	76.03
Ischemic heart disease		
Yes	2	1.65
No	119	98.35
Smoking status		
Yes	5	4.13
No	116	95.87
Family history of hepatic diseases		
Yes	1	0.83
No	120	99.17
Drug intake		
Yes	5	4.13
No	116	95.87
Cancer		
Yes	8	6.61
No	113	93.39
Fibrosis stages		
F0	11	9.09
F1/F2	89	73.55
F3/F4	21	17.36
FIB-4		
<1.30	60	49.59
1.30–2.67	31	25.62
>2.67	30	24.79
NFS		
<-1.455	68	56.20
-1.455 to 0.676	39	32.23
>0.676	14	11.57

FIB-4 = fibrosis-4 index, NFS = nonalcoholic fatty liver disease fibrosis score.

**Table 2:** Intergroup comparison of different degrees of fibrosis.

Variable	F0	F1–F2	F3–F4	*P*
Sex (male/female)	7/4	38/51	5/16	.384‡
Age (year)	30.18 ± 12.64	45.72 ± 13.65	55.24 ± 16.64	<.005*
BMI (kg/m^2^)	26.02 ± 3.86	25.80 ± 3.53	25.97 ± 4.38	.971*
PLT (×10^9^/L)	195.91 ± 62.44	193.65 ± 62.51	110.43 ± 43.86	<.005*
ALB (g/L)	46 (42.5–51.5)	43.5 (41.65–46)	39.2 (35.9–44.4)	<.005†
ALT (U/L)	120 (44.8–161.7)	71.5 (35.25–133.2)	31.3 (19.1–56)	.003†
AST (U/L)	49.8 (32–82)	41.4 (29.35–69.85)	42 (32–51.55)	.737†
GLU (mmol/L)	4.94 (4.23–5.68)	5.26 (4.65–6.01)	5.67 (5.12–6.86)	.056†
UA (μmol/L)	300 (253–391)	350 (304.5–407.5)	316 (294.5–439)	.211†
FIB-4	0.68 (0.49–0.92)	1.20 (0.77–2.07)	3.41 (2.55–6.52)	<.005†
NFS	-3.10 (-3.97–-2.11)	-1.95 (-3.14–-1.04)	0.55 (-0.48–1.41)	<.005†

ALB = albumin, ALT = alanine aminotransferase, AST = aspartate aminotransferase, BMI = body mass index, FIB-4 = fibrosis-4 index, GLU = glucose, NFS = nonalcoholic fatty liver disease fibrosis score, PLT = platelets, UA = uric acid.

* Analysis of variance.

† Kruskal–Wallis test.

‡ Chi-squared test.

The FIB-4 index and NFS results were compared with the liver fibrosis stages, as shown in Table 3. Of the 60 patients with an FIB-4 score < 1.30, 9 had F0 fibrosis stage, 50 had F1 or F2 fibrosis stage, and only one had advanced fibrosis (F3, F4). Fifteen of the 31 patients with FIB-4 scores > 2.67 had concordant results with liver biopsy. Of the 68 patients with NFS < −1.455, 10 had F0 fibrosis, 57 had F1 or F2 fibrosis, and only 1 had advanced fibrosis (F3, F4). Nine of the 14 patients with NFS > 0.676 had concordant results with liver biopsy.

**Table 3:** Comparison between fibrosis-4 index score and nonalcoholic fatty liver disease fibrosis score with liver biopsy results.

	Scheuer system			Total	*P*
	F0	F1, F2	F3, F4		
FIB-4 score					
<1.30	9 (15%)	50 (83.33%)	1 (1.67%)	60	<.005
1.30–2.67	1 (3.23%)	25 (80.64%)	5 (16.13%)	31	
>2.67	1 (3.33%)	14 (46.67%)	15 (50%)	30	
NFS					
<-1.455	10 (14.71%)	57 (83.82%)	1 (1.47%)	68	<.005
-1.455–0.676	0 (0%)	28 (71.79%)	11 (28.21%)	39	
>0.676	1 (7.14%)	4 (28.57%)	9 (64.29%)	14	

FIB-4 = fibrosis-4 index, NFS = NAFLD fibrosis score.

*P* < .05, Kruskal–Wallis test with continuous correction.

**Figure 1.**
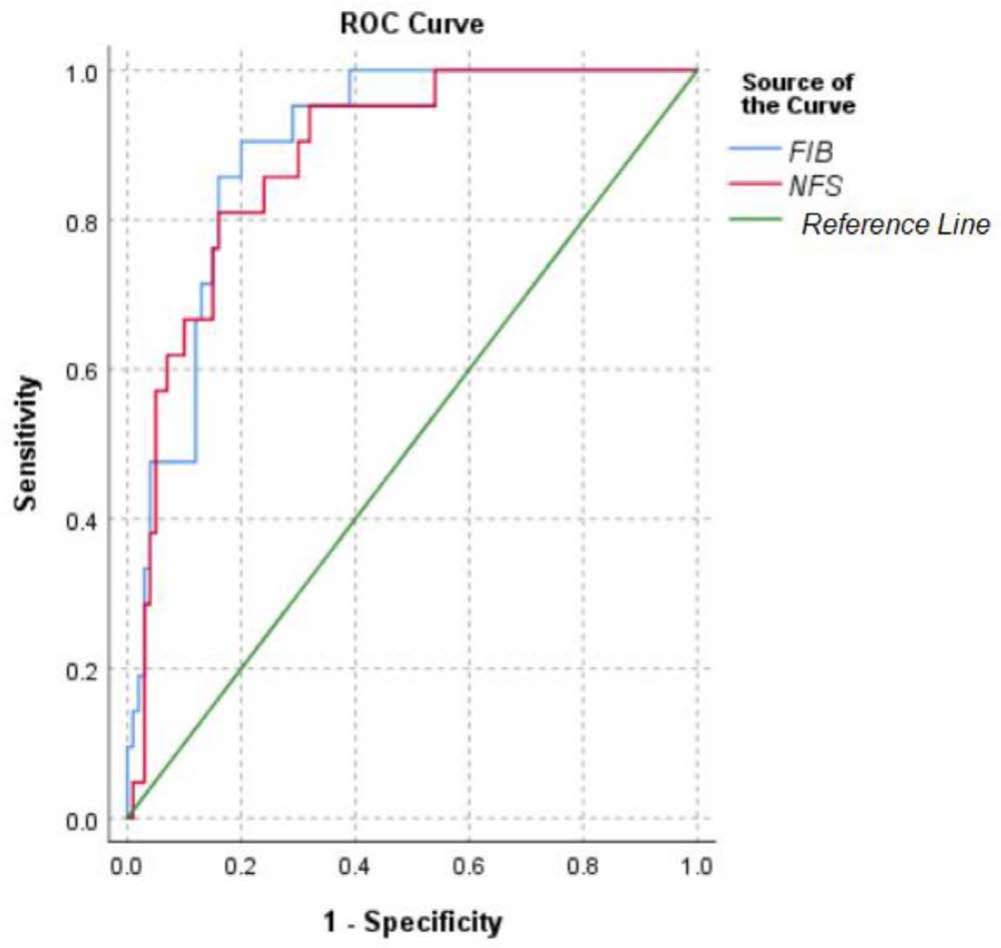
ROC curves of fibrosis-4 index and NFS. ROC = receiver operating characteristic, NFS = nonalcoholic fatty liver disease fibrosis score.

The patients were categorized into 2 groups based on their liver biopsy results. Group I comprised patients with minimal to mild fibrosis (F0–F2). Group II comprised patients with advanced fibrosis (F3–F4). The cutoff values of 2.67 for FIB-4 and 0.676 for NFS were set. The ROC curve analysis for FIB-4 and NFS revealed that the FIB-4 and NFS had an AUC of 0.895 (95% confidence interval [CI]: 0.836–0.954) and 0.882 (95% CI: 0.813–0.952), respectively. The ROC analysis demonstrated that FIB-4 and NFS could satisfactorily predict advanced fibrosis (AUC = 0.895, *P* < .001 for FIB-4 and AUC = 0.882, *P* < .001 for NFS) (Fig. 1).

To evaluate the effectiveness of FIB-4 and NFS as tools for detecting liver fibrosis, we calculated their sensitivity, specificity, positive predictive value, and NPV of FIB-4 and NFS for mild and advanced fibrosis (Table 4). For mild fibrosis, we used 1.30 a cutoff point, and any patient with an FIB-4 score > 1.30 was considered positive for mild fibrosis. Two cutoff points were used for advanced fibrosis: 1.30 and 2.67. We also used −1.455 as a cutoff point, and any patient with an NFS > −1.455 was considered positive for mild fibrosis. Two cutoff points were used for advanced fibrosis: −1.455 and 0.676. Research has shown that the FIB-4 index shows a sensitivity of 95.24% at the 1.30 cutoff point and a specificity of 85% at the 2.67 cutoff point, whereas the NFS index shows a sensitivity of 95.24% at the −1.455 cutoff point and a specificity of 95% at the 0.676 cutoff point.

**Table 4:** Performance of fibrosis-4 index and nonalcoholic fatty liver disease fibrosis score in detecting mild and advanced fibrosis.

	Cutoff	Sensitivity	Specificity	PPV	NPV
FIB-4 score					
Mild fibrosis (≥F1)	1.30	53.64%	81.82%	96.72%	15.00%
Advanced fibrosis (≥F3)	1.30	95.24%	59.00%	32.79%	98.33%
	2.67	71.43%	85.00%	50.00%	93.41%
NFS					
Mild fibrosis (≥F1)	-1.455	47.27%	90.91%	98.11%	14.71%
Advanced fibrosis (≥F3)	-1.455	95.24%	67.00%	37.74%	98.53%
	0.676	42.86%	95.00%	64.29%	88.79%

FIB-4 = fibrosis-4 index, NFS = nonalcoholic fatty liver disease fibrosis score, NPV = negative predictive value, PPV = positive predictive value.

Further analyses were performed to understand the cause of mismatch among the FIB-4 index, NFS, and liver biopsy results in some patients. We discovered that 26.67% of patients with minimal to mild fibrosis (F0, F1/F2) and an FIB-4 score > 2.67 had diabetes mellitus, and 40% had hypertension. Of the 51 patients with mild to advanced fibrosis (F1/F2, F3/F4) and FIB-4 scores < 1.3, 4 (7.84%) had diabetes mellitus and 6 (11.76%) had hypertension. Furthermore, 60% of the patients with minimal to mild fibrosis (F0, F1/F2) and an FIB-4 score > 0.676 had diabetes mellitus and 60% had hypertension. In contrast, of the 58 patients with mild to advanced fibrosis (F1/F2, F3/F4) and NFS < −1.455, 2 (3.45%) had diabetes mellitus and 9 (15.52%) had hypertension (Table 5).

**Table 5:** Variables associated with discordant biopsy, fibrosis-4 index and nonalcoholic fatty liver disease fibrosis score results.

	F0, F1/F2 (N = 100)		F1/F2,F3/F4 (N = 110)	
	FIB-4 > 2.67 (N = 15)	NFS > 0.676 (N = 5)	FIB-4 < 1.30 (N = 51)	NFS < -1.455 (N = 58)
Diabetes mellitus	26.67%	60%	7.84%	3.45%
Hypertension	40%	60%	11.76%	15.52%
Cancer diagnosis	33.33%	40%	1.96%	3.45%
Smoking status	13.33%	20%	3.92%	3.45%

FIB-4 = fibrosis-4 index, NFS = nonalcoholic fatty liver disease fibrosis score.

## 4. Discussion

We aimed to test whether the FIB-4 and NFS indices could successfully identify the level of liver fibrosis in patients with NAFLD. This study included 121 participants who were assessed using biopsy samples. Our analysis suggests that using the Scheuer system can effectively distinguish between the different severities of NAFLD, whereas methods such as the Fibro or APRI test may not be effective for the noninvasive detection of liver conditions. The distribution of liver fibrosis stages indicated that approximately 9.1% of the patients presented with minimal fibrosis (F0), and approximately 17.4% exhibited advanced fibrosis (F3 + F4); mild fibrosis (F1 + F2) was observed in most cases, accounting for 73.6% percent of those assessed.

In patients with NAFLD and liver fibrosis, the outcomes were compared using the FIB-4 index, NFS, and Schueuer scoring system. Of the 31 patients with an FIB-4 score between 1.30 and 2.67, 25 (80.64%) had similar results to liver biopsy (Scheuer F1–F2). Excluding patients with severe fibrosis (F3–F4), 30 had an FIB-4 score > 2.67; 15 (50%) of these had concordant results with the liver biopsy findings (Scheuer F3–F4), and 15 (50%) had contradictory results (F0–F1–F2). Of the 39 cases with an NFS between −1.455 and 0.676, 28 (71.79%) had similar results to those of the liver biopsy (Scheuer F1–F2). Excluding patients with severe fibrosis (F3–F4), 14 patients had an NFS > 0.676, of whom 9 (64.29%) had concordant results with the liver biopsy findings (Scheuer F3–F4), and 5 (35.71%) had discordant results (F0–F1–F2). These findings suggest that the NFS and FIB-4 may offer the best diagnostic performance for detecting mild fibrosis.

These results are consistent with those obtained by other authors. According to the China Guide for 2024, the critical FIB-4 and NFS values for cirrhosis are 2.67 and 0.676, respectively.^[18]^ Our research showed that the detected FIB-4 and NFS values of patients in the F3–F4 fibrosis group were higher than those of patients in the F0–F2 fibrosis group. The ROC curve analysis revealed an AUC of 0.895 (95% CI: 0.836–0.954) for FIB-4 and 0.882 (CI: 0.813–0.952) for NFS. The results for these 2 markers were statistically significant (*P* < .001). However, observation of the 2 AUCs showed that the FIB-4 index can predict severe liver fibrosis better than NFS. This may be related to differences in the laboratory parameters.

The NPV and sensitivity of the test (FIB-4) were 98.33% and 95.24%, respectively, with an advanced fibrosis cutoff point of 1.30, which allowed for the exclusion of nearly 95% of patients with advanced fibrosis. Using the upper cutoff value of 2.67, 50% of the patients with advanced fibrosis were correctly classified. However, the NPV and sensitivity of the test (NFS) were 98.53% and 95.24%, respectively, with an advanced fibrosis cutoff point of −1.455, which allowed for the exclusion of nearly 98% of patients with advanced fibrosis. Using an upper cutoff value of 0.676, 64.29% of patients with advanced fibrosis were correctly classified. Results from multiple studies^[19–22]^ indicate that the FIB-4 index has an NPV of 94.7%, sensitivity of 74.3%, and specificity of 80% for advanced fibrosis at a cutoff point of 1.30. However, with an upper limit of 2.67, the sensitivity and specificity for the diagnosis of significant fibrosis were 59% and 74%, respectively.

The primary goal of further analysis was to determine why some patients had liver biopsy results that differed from their corresponding FIB-4 and NFS indices. Obesity, type 2 diabetes mellitus, dyslipidemia, and NAFLD are interconnected metabolic disorders, sharing underlying molecular, biochemical, and immunological pathways.^[11,23]^ Our study found that individuals with minimal to mild fibrosis levels (F0–F2) who scored higher than 0.676 on the NFS showed an increased incidence of comorbidities such as diabetes or hypertension (approximately 60%) for each of the 2 variables, whereas in the FIB-4 test (>2.67), the 2 variables were approximately 26.67% and 40%, respectively. These findings are consistent with those of previous studies.^[24,25]^ While noninvasive methods for fibrosis assessment are recommended for patients with comorbidities such as diabetes and hypertension, their application is still surrounded by a degree of uncertainty. As some studies have indicated, NAFLD differs between patients with and without diabetes. From an anatomical and pathological perspective, there exist distinct differences between NAFLD in the presence and absence of type 2 diabetes mellitus. Studies have shown a high prevalence of NASH in patients with diabetes.^[26,27]^ Therefore, if a patient has comorbidities such as hypertension and diabetes, their corresponding FIB-4 and NFS test results may be affected, resulting in inconsistencies with their liver biopsy results. Additional research is warranted to elucidate the complexities associated with NAFLD in individuals presenting with risk factors pertinent to the condition.

This research offers a number of strengths while also acknowledging its inherent limitations. One of the study’s key strengths lies in the clarity of the NAFLD diagnosis and the precise staging of liver tissue fibrosis derived from biopsy assessments. The limitations include the following: the assessment of alcohol consumption was subjective, which may have influenced the size and quality of the study population. Additionally, its retrospective nature may have affected the sensitivity and specificity of the assessment tests. Finally, liver biopsy results may vary depending on the technique used by different specialists.

## 5. Conclusions

This study suggests that NFS and FIB-4 index are useful tools to guide further liver evaluation in patients with NAFLD in the early stages of fibrosis. However, further research is required to validate these findings.

## Acknowledgments

All the authors contributed considerably to this study. Mingxi Chen and Na Liu designed the study, Mingxi Chen and Chang Guo drafted the manuscript and analyzed the data, and Ke Ouyang was responsible for data collection and interpretation. All authors have reviewed, edited, and approved the final version of the manuscript. The authors acknowledge all participants in this study for their cooperation and contribution.

## Author contributions

**Conceptualization:** Na Liu.

**Data curation:** Mingxi Chen.

**Supervision:** Ke Ouyang.

**Validation:** Ke Ouyang.

**Writing – original draft:** Mingxi Chen, Chang Guo.

**Writing – review & editing:** Na Liu.
